# Knowledge, attitudes and practices of patients with chronic pharyngitis toward laryngopharyngeal reflux in Suzhou, China

**DOI:** 10.1186/s12889-023-17463-0

**Published:** 2023-12-19

**Authors:** Qiumin Zhang, Haiping Huang, Jiachen Li, Yuyu Niu, Peng Sun, Fuwei Cheng

**Affiliations:** Department of Otolaryngology, The First Affiliated Hospital of Soochow University, Suzhou, 215006 China

**Keywords:** Knowledge, Attitudes, Practices, Chronic pharyngitis, Laryngopharyngeal reflux, Questionnaire, Cross-sectional study

## Abstract

**Background:**

This study aimed to investigate the knowledge, attitudes and practices (KAP) of patients with chronic pharyngitis in Suzhou, China toward laryngopharyngeal reflux (LPR).

**Methods:**

This cross-sectional study was conducted in patients with chronic pharyngitis in Suzhou, China at the otolaryngology outpatient clinic of the First Affiliated Hospital of Soochow University between November, 2022, and May, 2023. Data was collected through a self-designed online questionnaire encompassing the sociodemographic characteristics and three dimensions of KAP. The questionnaire was administered using SoJump, and data were exported from this platform. Subsequently, statistical analysis, including Structural Equation Modeling, was performed using SPSS 22 software to evaluate the KAP scores.

**Results:**

A total of 487 valid questionnaires were collected, with 275 (56.35%) female patients. The mean score of KAP were 4.76 ± 2.93 (possible range: 0–11), 33.10 ± 4.46 (possible range: 8–40), 31.29 ± 6.04 (possible range: 8–40), respectively. Pearson’s correlation analysis showed significant positive correlations between knowledge and attitude dimensions (r = 0.413, *P* < 0.001), knowledge and practice dimensions (r = 0.355, *P* < 0.001), and attitude and practice dimensions (r = 0.481, *P* < 0.001). Structural equation modeling revealed that education exhibited positive effect on knowledge (β = 0.476, *P* < 0.001) and attitude (β = 0.600, *P* < 0.001), and having family history of chronic pharyngitis showed positive effect on knowledge (β = 0.580, *P* = 0.047), experienced with reflux symptoms showed positive effect on knowledge (β = 0.838, *P* = 0.001) and attitude (β = 0.631, *P* = 0.085). Moreover, knowledge showed positive effect on attitude (β = 0.555, *P* < 0.001) and practice (β = 0.351, *P* < 0.001). Attitude, in turn, showed positive effect on practice (β = 0.511, *P* < 0.001).

**Conclusion:**

Patients with chronic pharyngitis had inadequate knowledge, positive attitudes and suboptimal practices toward LPR. Education, family history of chronic pharyngitis, experienced with reflux symptoms might have effect on their KAP.

## Background

Chronic pharyngitis is a persistent inflammation of the pharyngeal mucosa, submucosa, and lymphoid tissues. It is a stubborn upper respiratory tract condition that is difficult to cure. Symptoms vary but often include discomfort such as foreign body sensation, burning, and speech issues [1, 2]. In the United States, there are 7,000,000 to 11,000,000 outpatient visits for chronic pharyngitis [3]. In China, the condition is prevalent due to dietary habits, environment, and antibiotic misuse, affecting around one-third of the population [4]. Laryngopharyngeal reflux (LPR) is closely linked to chronic pharyngitis and represents a significant underlying cause of this condition [5, 6]. LPR was initially proposed by Koufman as a distinctive variant of gastroesophageal reflux disease [7, 8]. LPR is defined as the retrograde flow of gastric-duodenal contents into the upper respiratory and digestive tract above the upper esophageal sphincter, affecting regions such as the nasopharynx, oropharynx, laryngopharynx, and larynx. This reflux event can lead to morphological changes in the upper respiratory and digestive tract and is associated with a diverse array of clinical symptoms and manifestations [9]. Research findings have revealed a notably high prevalence of LPR, with figures as substantial as 34.39% among residents in the UK [10], while approximately 28.12% of Chinese chronic pharyngitis patients are suspected to have LPR [11]. Clinically, patients predominantly present with symptoms such as hoarseness, cough, and throat pain, with gastrointestinal manifestations often being less prominent, thereby making it susceptible to oversight by both medical practitioners and patients [9]. LPR was initially proposed by Koufman as a distinctive variant of gastroesophageal reflux disease [7, 8]. The diagnosis of LPR typically involves a comprehensive approach, with primary care physicians, otolaryngologists, allergists, and gastroenterologists collaborating to evaluate patients’ medical history and clinical presentation [12–14].

Knowledge, attitudes and practices (KAP) research is a comprehensive survey method used to investigate the KAP of specific populations, including the general public, patients, and healthcare professionals, in particular domains [15]. Through KAP research, researchers gain a comprehensive understanding of the current status of a specific population’s knowledge and awareness of certain issues or diseases, their attitudes and inclinations, and their behaviors and practices in real-life situations. This method reveals cognitive biases, attitudinal tendencies, and behavioral habits, thereby providing crucial insights for improving public health efforts, formulating targeted health education programs, and optimizing clinical care [16].

Misdiagnosis of many LPR patients as having chronic pharyngitis has led to a lack of targeted treatment strategies for the underlying cause during the therapeutic process. Conventional use of antibiotics and other medications has proven ineffective in resolving the issue, leaving patients with inadequately alleviated symptoms, poor treatment outcomes, and even disease exacerbation [17]. Thus, this study aimed to investigate the KAP of patients with chronic pharyngitis in Suzhou, China toward LPR.

## Methods

### Study design and patients

This cross-sectional study was conducted at the otolaryngology outpatient clinic of the First Affiliated Hospital of Soochow University between November, 2022, and May, 2023. The study included individuals diagnosed with chronic pharyngitis who fulfilled specific inclusion criteria, namely, being patients in the otorhinolaryngology outpatient department, aged over 18 years, and experiencing repeated pharyngeal paraesthesia, throat itch, and dry pharynx for more than two months. Exclusion criteria comprised patients with organic throat diseases such as tonsil hypertrophy, chronic tonsillitis, and benign and malignant throat tumors. This study was conducted in accordance with ethical principles and guidelines. The research protocol was reviewed and approved by the ethical approval of the Institutional Ethics Committee of the First Affiliated Hospital of Soochow University before commencement. Informed consent was obtained from all participants, and they were assured of confidentiality and anonymity throughout the study. Any potential conflicts of interest were disclosed and managed appropriately.

### Questionnaire

The questionnaire design was based on the *Standardized diagnosis and treatment of laryngopharyngeal reflux diseases* and *The BMJ clinical practice website*: http://bestpractice.bmj.com. The initial draft underwent revisions with inputs from three experts, followed by a small-scale distribution (30 copies) for feedback. The reliability and validity test yielded a Cronbach’s α coefficient of 0.855, indicating good internal consistency. The final questionnaire, presented in Chinese, consisted of four dimensions comprising a total of 42 items. These dimensions included sociodemographic characteristics (15 items), knowledge (11 items), attitudes (8 items), and practices (8 items). Points were assigned based on the number of options for each item. The knowledge dimension had correct answers assigned 1 point, while incorrect or unclear answers received 0 points. The attitude and practice dimensions employed a five-point Likert scale, ranging from very positive (5 points) to very negative (1 point).

Three trained research assistants recruited these confirmed patients through outpatient follow-up visits and patient WeChat groups, and free laryngopharyngeal reflux relevant inspections were given to encourage participation. Participants who met the inclusion criteria were sent the questionnaire electronically through WeChat groups. The online questionnaire based on the “*SoJump* (https://www.wjx.cn/)” application of WeChat was used for the survey, and a QR code was generated to allow the data collection through WeChat. Participants log in by scanning the QR code sent by WeChat, and then complete the questionnaire. In addition, research assistants were provided detailed full process training, including explaining the contents of the questionnaire to responders, procedures for sending and collecting questionnaires, rules for filling questionnaires, and data export, etc. During the questionnaire response, clarifying questions/items were performed by research assistants to ensure that respondents fully understand the meaning of the questionnaire and the intent of this survey. To guarantee the quality and completeness of the questionnaire survey, each IP address could only submit the questionnaire once, and all questions in the questionnaire were mandatory, though no repeat questions implemented. After questionnaire collection, the completeness, internal coherence, and reasonableness of all questionnaires were checked by the investigators. Moreover, before statistical analysis, we performed rigorous data cleaning to obtain valid questionnaires. The cleaning delete criteria were: repeated questionnaires, abnormal data (for example, the age value is too large or too small, the options are not logical, all the same options are selected, etc.), incomplete data, and the answer time is less than 90 s.

### Statistical analysis

Sample size was calculated using the formula for cross-sectional studies: α = 0.05, n= (Z_ (1-α/2)/δ)^2×p× (1-p) where Z_ (1-α/2) = 1.96 when α = 0.05, the assumed degree of variability of *p* = 0.5 maximises the required sample size, and δ is admissible error (which was 5% here). The theoretical sample size was 480 which includes an extra 20% to allow for subjects lost.

The statistical analysis were performed using SPSS 22 software (IBM Corp., Armonk, N.Y., USA). Descriptive analysis was applied to the demographic data and KAP scores of patients. Continuous variables conforming to normal distribution were presented as Mean ± SD and compared using Student’s t-test or ANOVA. Categorical data were expressed as n (%). Pearson’s correlation analysis was employed to assess correlations between variables. Structural equation modeling (SEM) was utilized to explore the path relationship between KAP scores and general information. Model fit was evaluated using root mean square error of approximation (RMSEA), incremental fit index (IFI), Tucker–Lewis index (TLI), and comparative fit index (CFI). A two-sided *P*-value less than 0.05 was considered statistically significant.

## Results

Initially, 500 questionnaires were collected. After excluded for completely duplicated options (4 cases), patients under 18 years old (3 cases), abnormal value in reflux option (1 case), and all identical responses in KAP dimensions (5 cases), 487 valid questionnaires remained for analysis. Among them, 275 (56.35%) of the patients were female. The mean score of knowledge, attitude and practice were 4.76 ± 2.93 (possible range: 0–11), 33.10 ± 4.46 (possible range: 8–40), 31.29 ± 6.04 (possible range: 8–40), respectively. Additionally, females (*p* = 0.035), and patients residing in urban areas (*p* = 0.005) were more likely to have higher knowledge scores, while education significantly influenced knowledge scores (*p* < 0.001). Similarly, a higher monthly per capita income was linked to a higher likelihood of higher knowledge scores (*p* < 0.001). Unmarried patients were more likely to have greater knowledge scores (*p* = 0.035). Smoking and drinking habits had a significant impact on attitude scores (*p* < 0.001 and *p* = 0.002, respectively), with non-smokers and non-drinkers being more likely to display more favorable attitudes. Family history of chronic pharyngitis significantly influenced attitude scores (*p* < 0.001), with patients having a family history being more likely to have higher scores (Table 1).

**Table 1:** Sociodemographic characteristics

	N (%)/mean ± SD	K		A		P	
		Mean ± SD	p	Mean ± SD	p	Mean ± SD	p
**Total**	487	4.76 ± 2.93		33.10 ± 4.46		31.29 ± 6.04	
**Gender**			0.470		0.035		0.615
Male	213 (43.65)	4.65 ± 2.99		32.61 ± 4.62		31.14 ± 6.04	
Female	275 (56.35)	4.84 ± 2.88		33.47 ± 4.30		31.41 ± 6.05	
**Age**	39.21 ± 11.48	-	-	-	-	-	-
**Residence**			0.005		0.010		0.001
Urban	360 (73.92)	4.98 ± 2.92		33.41 ± 4.45		31.82 ± 5.87	
Non-urban	127 (26.08)	4.13 ± 2.87		32.22 ± 4.37		29.81 ± 6.28	
**Education**			< 0.001		< 0.001		< 0.001
Junior high school or below	108 (22.18)	3.72 ± 2.76		31.44 ± 4.36		28.82 ± 6.51	
High school/technical secondary school	91 (18.69)	4.25 ± 3.11		31.91 ± 4.53		29.96 ± 6.11	
College/Bachelor	241 (49.49)	5.30 ± 2.82		34.00 ± 4.19		32.53 ± 5.42	
Master or above	47 (9.65)	5.30 ± 2.72		34.57 ± 4.25		33.21 ± 5.52	
**Employment**			0.756		0.164		0.052
Employed	352 (72.28)	4.78 ± 2.95		33.27 ± 4.48		31.62 ± 5.91	
Unemployed	135 (27.72)	4.69 ± 2.87		32.64 ± 4.39		30.44 ± 6.29	
**Monthly per capita income, Yuan**			0.001		0.001		< 0.001
< 5,000	165 (33.88)	4.16 ± 2.87		32.08 ± 4.35		29.04 ± 6.29	
5,000–10,000	166 (34.09)	4.71 ± 2.83		33.15 ± 4.19		31.83 ± 5.61	
10,000–20,000	103 (21.15)	5.32 ± 3.16		34.09 ± 4.72		32.74 ± 5.74	
> 20,000	53 (10.88)	5.66 ± 2.56		34.19 ± 4.47		33.85 ± 4.85	
**Marital status**			0.035		0.008		0.116
Unmarried	107 (21.97)	5.40 ± 2.81		33.92 ± 4.55		32.05 ± 6.04	
Married	355 (72.90)	4.58 ± 2.95		32.73 ± 4.38		30.96 ± 6.04	
Other	25 (5.13)	4.52 ± 2.83		34.80 ± 4.47		32.80 ± 5.67	
**Smoking**			0.408		< 0.001		0.171
Never	382 (78.28)	4.80 ± 2.91		33.51 ± 4.25		31.48 ± 5.97	
Used to smoke	52 (10.66)	4.25 ± 3.00		30.71 ± 5.32		29.81 ± 5.98	
Still smoke now	54 (11.07)	4.91 ± 2.99		32.50 ± 4.27		31.41 ± 6.46	
**Drinking**			0.098		0.002		0.332
Never	325 (66.60)	4.95 ± 2.86		33.60 ± 4.23		31.57 ± 6.06	
Used to drink	78 (15.98)	4.26 ± 3.15		31.94 ± 4.70		30.95 ± 6.05	
Still drink now	85 (17.42)	4.45 ± 2.94		32.22 ± 4.80		30.55 ± 5.95	
**Medical insurance**							
Urban Employee Basic Medical Insurance	409 (83.81)	4.82 ± 2.92		33.29 ± 4.39		31.51 ± 5.88	
The new rural cooperative medical insurance	51 (10.45)	3.72 ± 2.91		31.78 ± 4.41		29.34 ± 5.74	
Urban Residents Basic Medical Insurance	19 (3.89)	5.63 ± 3.15		34.26 ± 4.33		31.16 ± 8.22	
Retired Cadres Medical Insurance	1 (0.20)	8.00		31.00		26.00	
Commercial insurance	29 (5.94)	5.48 ± 2.72		34.55 ± 4.02		31.24 ± 5.84	
None	17 (3.48)	5.29 ± 2.82		31.29 ± 5.44		30.47 ± 7.45	
**Course of chronic pharyngitis**			0.266		0.657		0.292
< 1 years	255 (52.25)	4.52 ± 2.96		33.04 ± 4.65		31.70 ± 6.16	
1–3 years	112 (22.95)	5.06 ± 3.03		32.79 ± 4.27		31.12 ± 5.83	
3–5 years	49 (10.04)	5.18 ± 2.68		33.53 ± 4.11		31.27 ± 5.57	
> 5 years	72 (14.75)	4.82 ± 2.80		33.50 ± 4.31		30.17 ± 6.20	
**Experienced reflux symptoms**			0.002		0.013		0.249
Yes	223 (45.79)	5.20 ± 2.96		33.64 ± 4.28		31.64 ± 5.90	
No	264 (54.21)	4.38 ± 2.85		32.64 ± 4.56		31.00 ± 6.15	
**Underlying or chronic diseases**			0.910		0.291		0.604
Yes	98 (20.12)	4.79 ± 2.97		32.67 ± 4.72		31.01 ± 6.05	
No	389 (79.88)	4.75 ± 2.92		33.21 ± 4.39		31.37 ± 6.04	
**Family history of chronic pharyngitis**			< 0.001		0.190		0.173
Yes	124 (25.41)	5.43 ± 2.83		33.70 ± 4.29		32.06 ± 5.73	
No	217 (44.47)	4.88 ± 2.93		32.79 ± 4.29		31.26 ± 6.17	
Unclear	147 (30.12)	4.01 ± 2.85		33.04 ± 4.81		30.69 ± 6.06	
**History of throat surgery**			0.441		0.839		0.903
Yes	17 (3.48)	5.29 ± 2.89		32.88 ± 4.12		31.12 ± 7.44	
No	471 (96.52)	4.74 ± 2.93		33.11 ± 4.47		31.30 ± 5.99	

A total of 198 patients (40.66%) exhibited a precise understanding of the concept of LPR, 215 (44.15%) patients correctly identified gastrointestinal disease as a high-risk factor based on their knowledge, and 275 (56.47%) patients accurately recognized the clinical symptoms of reflux laryngitis. Furthermore, 177 (36.34%) patients were knowledgeable about the recommended diagnostic approach involving 24-hour larynopharyngeal pH surveillance, while 222 (45.59%) patients understood the necessity of a minimum six-month drug intervention for treatment. Lastly, 99 (20.33%) patients were aware of the improved prognosis and reduced likelihood of recurrence with proper medical attention and adherence to the treatment plan (Table 2).

**Table 2:** Distribution of Knowledge

Knowledge	Correct N (%)
1. Laryngopharyngeal reflux refers to the reflux of gastric contents to the throat above the upper esophageal sphincter, irritating the throat mucosa and triggering a series of inflammatory responses.	198 (40.66)
2. Obesity can cause increased abdominal pressure and enhance the reflux, but obesity is not a high-risk factor of laryngopharyngeal reflux.	26 (5.34)
3. Gastrointestinal disease is a high-risk factor of laryngopharyngeal reflux.	215 (44.15)
4. The clinical symptoms of reflux laryngitis include hoarseness, laryngeal obstruction, pharyngeal paraesthesia, and throat clearing, accompanied with acid reflux and heartburn, etc.	275 (56.47)
5. There is no recognized etiology of reflux laryngitis, but its is mostly closely related to gastrointestinal diseases, medications, lifestyle habits, etc. The gold standard for diagnosis is 24-hour larynopharyngeal PH surveillance.	177 (36.34)
6. Smoking, alcoholism, preference for strong tea, coffee, high-fat diet, and foods that easily promote increased gastric acid secretion can increase the risk of reflux laryngitis.	313 (64.27)
7. Someone with hoarseness, laryngeal obstruction, pharyngeal paraesthesia and throat clearing, accompanied with acid reflux, heartburn and other symptoms should go to the internal medicine department in time.	4 (0.82)
8. Patients should reduce the intake of high-sugar and high-fat (such as chocolate, cheese), acidic foods (such as citrus, carbonated drinks), spicy foods, and dioscillary foods.	370 (75.98)
9. Patients should quit smoking, alcohol, lose weight and lower fat, reduce life pressure, and ensure adequate sleep.	417 (85.63)
10. The treatment course of reflux laryngitis is long, requiring at least half a year of drug intervention.	222 (45.59)
11. Reflux laryngitis is not easy to recur after treatment.	99 (20.33)

It is evident that 209 (42.92%) patients strongly agreed, while 191 (39.22%) agreed with the proposition that patients suffering from chronic pharyngitis are susceptible to reflux laryngitis and necessitate vigilant measures for prevention. Moreover, 182 (37.37%) patients strongly agreed and 217 (44.56%) agreed that there is a serious lack of understanding among people regarding the causes, triggers, harms, and treatment of reflux laryngitis. Additionally, 200 (41.07%) patients strongly agreed and 209 (42.92%) agreed that suspected symptoms of reflux laryngitis require attention to rule out other more serious diseases. Furthermore, 206 (42.30%) patients expressed a positive attitude towards the comprehensive management of reflux laryngitis, believing that treatment should not rely solely on drugs, but also involve dietary and lifestyle changes. In contrast, a considerable number of patients held negative attitudes (N) towards certain aspects of reflux laryngitis management. For instance, 62 (31.01%) patients disagreed and 169 (34.70%) strongly agreed with the idea of not quitting smoking and drinking for reflux laryngitis (Table 3).

**Table 3:** Distribution of attitude

Attitude	Strongly agree	Agree	Neutrality	Disagree	Strongly disagree
1. I think that reflux laryngitis is irrelevant, which can not affect normal life.	20 (4.11)	20 (4.11)	73 (14.99)	245 (50.31)	129 (26.49)
2. I think that patients with chronic pharyngitis are prone to reflux laryngitis and need vigilance and prevention.	209 (42.92)	191 (39.22)	69 (14.17)	11 (2.26)	7 (1.44)
3. I think people have a serious lack of understanding of the causes, triggers, harms and treatment of reflux laryngitis.	182 (37.37)	217 (44.56)	73 (14.99)	10 (2.05)	5 (1.03)
4. I think that the suspected symptoms of reflux laryngitis need to be paid attention to to rule out other more serious diseases.	200 (41.07)	209 (42.92)	65 (13.35)	10 (2.05)	3 (0.62)
5. I think that the treatment of reflux laryngitis cannot be managed by drugs alone, but by changing diet and lifestyle habits.	206 (42.30)	197 (40.45)	68 (13.96)	11 (2.26)	5 (1.03)
6. I think it is important to follow the doctor’s advice and use medication regularly.	280 (57.49)	165 (33.88)	36 (7.39)	4 (0.82)	2 (0.41)
7. I am reluctant to quit smoking and drinking for reflux laryngitis.	62 (12.73)	49 (10.06)	56 (11.50)	151 (31.01)	169 (34.70)
8. I am willing to lose weight and lose fat.	221 (45.38)	198 (40.66)	58 (11.91)	9 (1.85)	1 (0.21)

It is notable that 262 (53.80%) patients expressed their intent to proactively address reflux laryngitis, while 186 (38.19%) patients intended to reduce their consumption of strong tea, coffee, and soda. Moreover, 184 (37.78%) patients expressed their intention to adopt habits such as eating small and frequent meals, staying upright after meals, and engaging in moderate exercise. Additionally, a considerable number of patients indicated their willingness to observe habits like fasting 2–3 h before bedtime (173, 35.52%) and avoiding staying up late and overworking (180, 36.96%). Furthermore, 164 (33.68%) patients mentioned their involvement in stress-relieving activities, and 473 (97.13%) patients conveyed their intention to prioritize cessation smoking and alcohol consumption. Moreover, 147 (30.18%) patients stated their readiness to raise awareness about the treatment and prevention of reflux laryngitis through various means such as WeChat groups, the internet, and popular science lectures (Table 4).

**Table 4:** Distribution of practice

Practice	Always	Usually	Sometimes		Occasionally	Never
1. I will actively treat reflux laryngitis.	262 (53.80)	110 (22.59)	82 (16.84)		24 (4.93)	9 (1.85)
2. I will drink less or no strong tea, coffee, soda, etc.	186 (38.19)	105 (21.56)	88 (18.07)		63 (12.94)	45 (9.24)
3. I will develop the habit of eating small and frequent meals, staying upright after meals, and exercising a small amount.	184 (37.78)	117 (24.02)	131 (26.90)		45 (9.24)	10 (2.05)
4. I will keep the habit of fasting 2–3 h before bedtime.	173 (35.52)	115 (23.61)	137 (28.13)		53 (10.88)	9 (1.85)
6. I will take care to avoid staying up late and overworking.	180 (36.96)	130 (26.69)	129 (26.49)		35 (7.19)	13 (2.67)
7. I participate in activities that relieve the stress of life.	164 (33.68)	93 (19.10)	154 (31.62)		47 (9.65)	29 (5.95)
8. I will raise awareness of the treatment and prevention of reflux laryngitis through WeChat groups, the Internet, popular science lectures, etc.	147 (30.18)	69 (14.17)	138 (28.34)		66 (13.55)	67 (13.76)
	Yes			No		
5. I will pay attention to quitting smoking and drinking.	473 (97.13)			14 (2.87)		

**Fig. 1:**
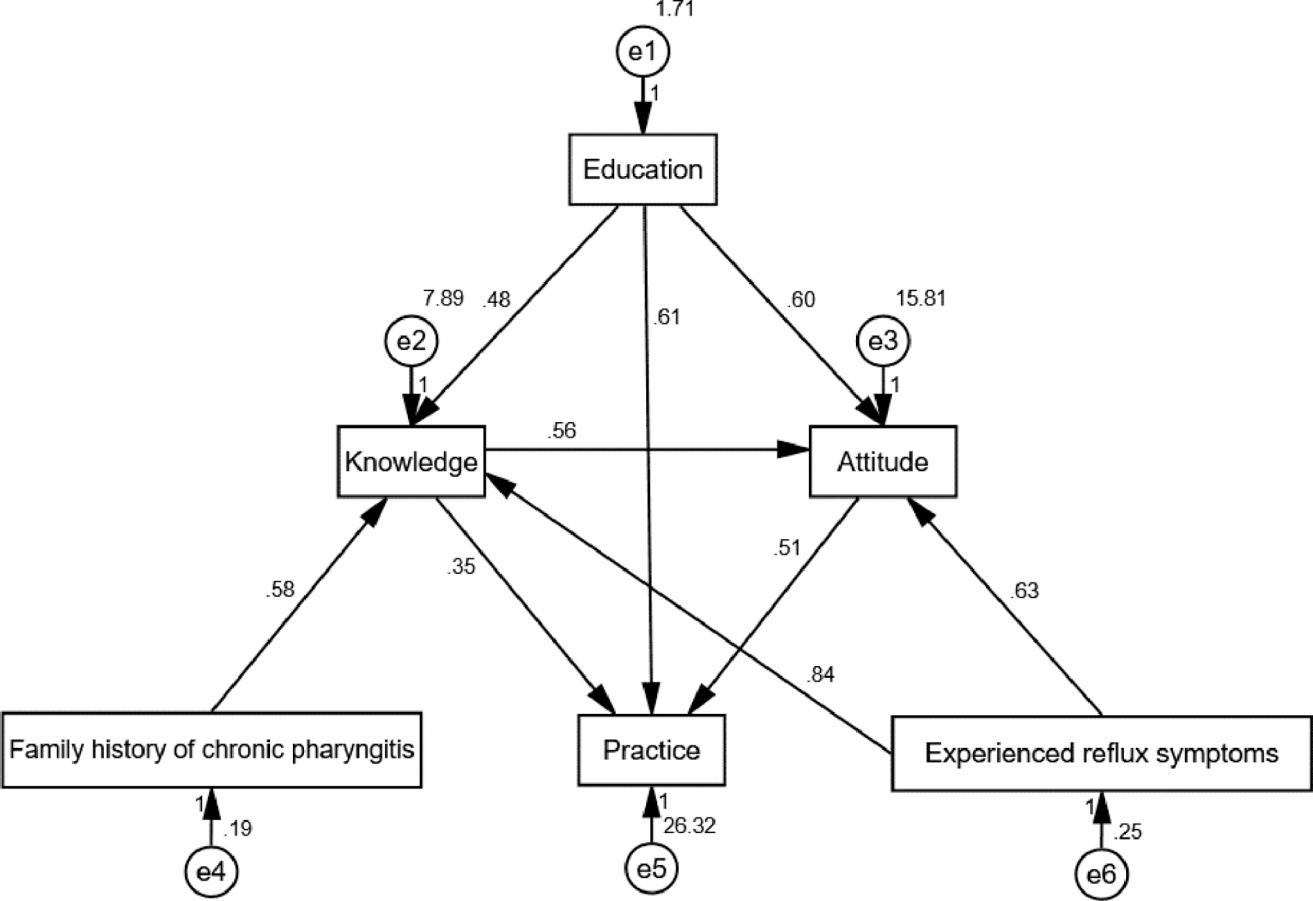
Structural Equation Modeling

Pearson’s correlation analysis showed significant positive correlations between knowledge and attitude dimensions (r = 0.413, *P* < 0.001), knowledge and practice dimensions (r = 0.355, *P* < 0.001), and attitude and practice dimensions (r = 0.481, *P* < 0.001) (Table 5). Structural equation modeling revealed that, education exhibited positive effect on knowledge (β = 0.476, *P* < 0.001) and attitude (β = 0.600, *P* < 0.001), and having family history of chronic pharyngitis showed positive effect on knowledge (β = 0.580, *P* = 0.047), experienced with reflux symptoms showed positive effect on knowledge (β = 0.838, *P* = 0.001) and attitude (β = 0.631, *P* = 0.085). Moreover, knowledge showed positive effect on attitude (β = 0.555, *P* < 0.001) and practice (β = 0.351, *P* < 0.001). Attitude, in turn, showed positive effect on practice (β = 0.511, *P* < 0.001) (Fig. 1; Table 6). The fit indices collectively, including CMIN/DF = 3.299 (Ref, excellent: 1–3, good: 3–5), RMSEA = 0.069 (Ref, good: <0.08), IFI = 0.957 (Ref, good: >0.8), TLI = 0.889 (Ref, good: >0.8), and CFI = 0.956 (Ref, good: >0.8), suggested that the hypothesized model aligns well with the observed data, demonstrating a satisfactory fit for the SEM analysis in this study (Table 7).

**Table 5:** Correlation analysis

	Knowledge	Attitude	Practice
Knowledge	1		
Attitude	0.413 (*P* < 0.001)	1	
Practice	0.355 (*P* < 0.001)	0.481 (*P* < 0.001)	1

**Table 6:** Structural Equation Modeling

			Β	P
K	<---	Education	0.476	< 0.001
K	<---	Family history of chronic pharyngitis	0.580	0.047
K	<---	Experienced reflux symptoms	0.838	0.001
A	<---	K	0.555	< 0.001
A	<---	Experienced reflux symptoms	0.631	0.085
A	<---	Education	0.600	< 0.001
P	<---	K	0.351	< 0.001
P	<---	A	0.511	< 0.001
P	<---	Education	0.607	0.001

**Table 7:** Goodness of fit

Indicators	Reference standards	Measured results
CMIN/DF	1–3 means excellent and 3–5 means good	3.299
RMSEA	< 0.08 means good	0.069
IFI	> 0.8 means good	0.957
TLI	> 0.8 means good	0.889
CFI	> 0.8 means good	0.956

## Discussion

This study suggested that patients with chronic pharyngitis showed inadequate knowledge, positive attitudes, and suboptimal practices toward LFR. Education, family history of chronic pharyngitis, experienced with reflux symptoms might have effect on their KAP. These findings might be benefit for the management of LPR among patients with chronic pharyngitis.

The study’s findings regarding patients with chronic pharyngitis reveal a notable disparity among their KAP related to LPR. Specifically, the observed inadequacy in knowledge, despite predominantly positive attitudes, highlights a potential gap in understanding among this patient population. Furthermore, the presence of suboptimal practices signifies a disconnection between positive attitudes and practical application. Gender differences in health-related knowledge have been consistently observed in previous studies. Females tend to seek health information more frequently and are more likely to engage in health-related discussions compared to males [17]. Accordingly, it is not surprising that in our study, females exhibited higher knowledge scores toward LPR than males. The disparities between urban and rural areas in terms of health access and awareness have been well-documented [18, 19]. Urban populations generally have better access to healthcare facilities, education resources, and awareness campaigns, which may explain why patients residing in urban areas in our study showed higher knowledge scores on reflux laryngitis.

The positive association between education and health literacy has been extensively reported [20, 21]. Individuals with higher education tend to have better health knowledge and comprehension of health-related materials. This aligns with our findings that patients with higher education exhibited greater knowledge scores on LPR. Socioeconomic status is a crucial determinant of health outcomes [22]. Higher income levels are associated with better access to healthcare services and engagement in health-seeking behaviors. Hence, it is not surprising that patients with a higher monthly per capita income in our study displayed higher knowledge scores. Marital status has also been associated with differences in health behaviors and engagement [23]. Married individuals may prioritize family responsibilities over personal health, which can lead to differences in health-related practices. This aligns with our findings that unmarried patients had greater knowledge scores on LPR.

Positive attitudes towards health conditions have been linked to better adherence to treatment and preventive practices [24]. In our study, patients who expressed positive attitudes towards reflux laryngitis were more willing to engage in recommended practices. Negative attitudes towards behavior change, such as quitting smoking and drinking, can be influenced by social and cultural factors [25, 26]. Cultural norms and peer influence play a significant role in shaping health-related behaviors. Therefore, the negative attitudes towards smoking and drinking cessation in our study might be attributed to cultural factors that need to be addressed in intervention programs.

The study’s results underscore differing levels of engagement among patients in managing reflux laryngitis, revealing opportunities for improvement in adopting consistent treatment and lifestyle adjustments. To enhance patient care practices, focused educational efforts, encouragement of healthier habits, provision of clear management guidelines, support for patient-doctor collaboration, and the promotion of informed decision-making empower patients to effectively manage reflux laryngitis, ultimately leading to better treatment outcomes [27, 28]. The correlation and structural equation modeling analyses confirmed the associations among KAP. Patients with higher knowledge scores were more likely to exhibit positive attitudes and engage in recommended practices. Attitudes also positively influenced practices, suggesting that fostering positive attitudes towards reflux laryngitis could lead to better adherence to management strategies [29].

This study had limitations. Firstly, the study design was cross-sectional, which means that causality cannot be established. The data collected at a single point in time may not provide a complete understanding of the dynamic relationships among KAP. Secondly, the data were gathered through self-reporting using questionnaires, which could introduce response bias and may not accurately reflect patients’ actual behaviors or experiences. Thirdly, there might be social desirability bias [30], where patients provide responses they believe are socially acceptable rather than their true beliefs or actions. Despite these constraints, the study’s substantial sample size and comprehensive exploration of KAP dimensions, along with the inclusion of sociodemographic characteristics, ˙contribute to the existing literature. These strengths underscore the study’s importance, despite its inherent limitations.

In conclusion, patients with chronic pharyngitis exhibited insufficient knowledge, positive attitudes, and suboptimal practices toward LPR. Education, family history of chronic pharyngitis, experienced with reflux symptoms might have effect on their KAP. The findings highlight the importance of targeted educational interventions to enhance knowledge and practice for the improvement of the management of LPR among patients with chronic pharyngitis.

## Data Availability

All data generated or analysed during this study are included in this published article.

## List of abbreviations

KAP: Knowledge, attitudes and practices
LPR: Laryngopharyngeal reflux
